# Managing Stroke Risk in Moyamoya Disease With Paroxysmal Atrial Fibrillation: A Case Report

**DOI:** 10.7759/cureus.85721

**Published:** 2025-06-10

**Authors:** Ahmed Kazi, Aniq Saleem, Abeera Akram

**Affiliations:** 1 Internal Medicine, Saint Francis Hospital, Hartford, USA; 2 Medicine, Fatima Memorial Hospital, Lahore, PAK; 3 Cardiology, University of Connecticut, Farmington, USA

**Keywords:** anticoagulation, antiplatelet therapy, atrial fibrillation, catheter ablation, cerebrovascular disease, collateral vessels, coronary artery disease, hypertension, moyamoya disease, stroke

## Abstract

Moyamoya disease is a progressive cerebrovascular disorder characterized by the narrowing and occlusion of arteries at the base of the brain, leading to the development of collateral vessels, which increases the risk of both ischemic and hemorrhagic strokes. The presence of atrial fibrillation (AF), particularly in a patient with Moyamoya disease, complicates the management strategy due to the dual risks of thromboembolism and hemorrhage. We present a case of a 40-year-old female with a history of unilateral right-brain Moyamoya disease, treated surgically, and with two prior strokes, left carotid stenosis, and hypertension. She was undergoing evaluation for arrhythmia with an implantable loop recorder (ILR) in place and presented to the ED with palpitations. Her ILR was interrogated, revealing two episodes of paroxysmal AF. Heart rate control was achieved with beta-blockers. Due to an elevated CHA₂DS₂-VASc score of 5, anticoagulation was recommended. However, there was concern about the increased risk of spontaneous cerebral hemorrhage in the setting of Moyamoya disease. After consulting with a neurologist and weighing the risks versus benefits, the patient was started on apixaban. Regarding the management of her AF, the patient expressed interest in pursuing catheter ablation; however, the procedure had not been performed at the time of this report. This case highlights the complexity of managing patients with both Moyamoya disease and paroxysmal AF. Treatment should be individualized, considering the benefits and risks of anticoagulation versus antiplatelet therapy, with an emphasis on stroke prevention while minimizing the risk of bleeding.

## Introduction

Moyamoya disease is a progressive cerebrovascular disorder characterized by the narrowing and occlusion of the terminal carotid arteries at the base of the brain, leading to the development of collateral vessels that resemble a "puff of smoke" on angiography [1]. This abnormal vascular network increases the risk of both ischemic and hemorrhagic strokes [2]. The presence of atrial fibrillation (AF), particularly in a patient with Moyamoya disease, complicates the management strategy due to the dual risks of thromboembolism (from AF) and hemorrhage (due to the fragile collateral vessels in Moyamoya disease) [3].

The choice of anticoagulation in such cases becomes challenging because standard anticoagulation therapy used in AF, such as direct oral anticoagulants (DOACs) or warfarin, may increase the risk of intracranial hemorrhage [4], which is a significant concern in Moyamoya disease. On the other hand, the risk of ischemic stroke due to thromboembolism in the setting of AF cannot be ignored. There is limited evidence in the literature regarding the optimal antithrombotic strategy in patients with concurrent Moyamoya disease and AF. Most decisions are individualized and guided by multidisciplinary collaboration, assessing the patient's stroke and bleeding risk factors.

## Case presentation

A 40-year-old female with a past medical history of unilateral right-brain Moyamoya disease with subsequent surgical treatment, two prior strokes, left carotid stenosis, hypertension, and an implantable loop recorder (ILR) in place for arrhythmia workup, presented to the ED with palpitations. The patient was lying in bed at home when she began experiencing palpitations associated with shortness of breath. Emergency medical services (EMS) were called, and she was found to be in AF with a heart rate of 170 bpm. En route to the hospital, the patient became hypotensive. She was emergently given lorazepam and cardioverted to sinus rhythm with 125 joules. Upon arrival at the ED, she complained of chest pain localized to the site of cardioversion.

On physical examination, she was a morbidly obese woman with a blood pressure of 167/107 mmHg and a heart rate of 104 bpm. Neck examination revealed no carotid bruits. Cardiac examination showed a normal, regular S1-S2 without murmurs. There was no edema on lower extremity examination.

Laboratory workup was unremarkable, with normal troponin T, ProBNP, TSH, and a negative beta-HCG. An EKG demonstrated normal sinus rhythm at 89 bpm with no significant abnormalities. A chest X-ray was negative for acute cardiopulmonary disease. Interrogation of her ILR revealed two episodes of paroxysmal AF, the most recent correlating with the time of presentation.

**Figure 1:**
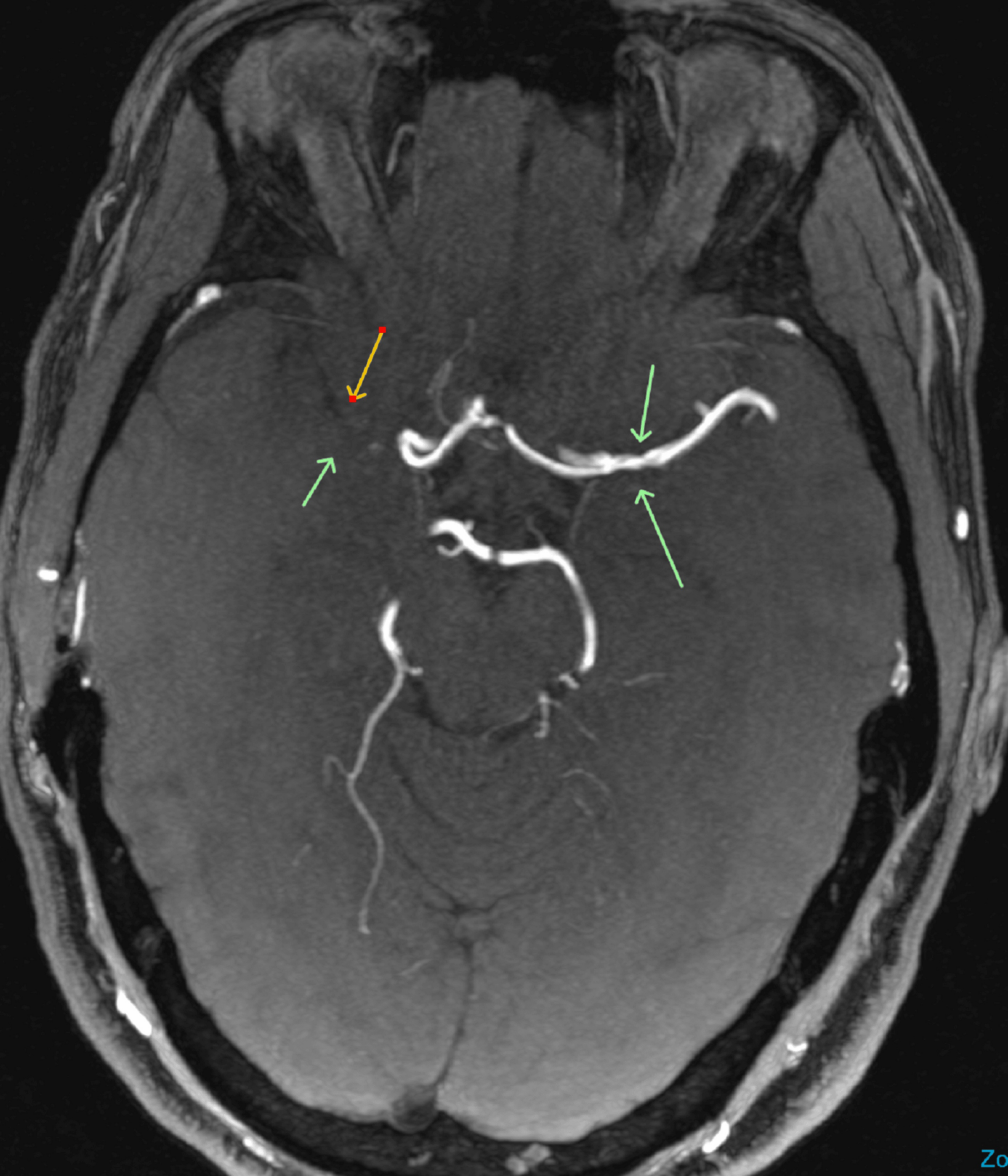
MRA of the brain showing narrowed vasculature and diminished anterior circulation consistent with Moyamoya disease. MRA: Magnetic Resonance Angiography.

The patient described her chest pain as sharp, intermittent, non-radiating, and localized at the site of cardioversion. The differential diagnosis included non-cardiac musculoskeletal pain or costochondritis. Given the negative troponin and normal EKG, acute coronary syndrome (ACS) was considered unlikely. She was discharged home with a plan to follow up with a cardiologist. The next day, the patient was evaluated by a cardiologist and started on metoprolol for rate control. Given her elevated CHA₂DS₂-VASc score of 5 (due to prior stroke, hypertension, vascular disease, and female sex), anticoagulation was recommended. However, there was concern about the increased risk of spontaneous cerebral hemorrhage in the context of Moyamoya disease (Figure 1).

After discussion with a neurologist and weighing the risks versus benefits, the patient was started on apixaban. She was also referred to an electrophysiologist for consideration of a left atrial appendage closure (LAAC) device, given the long-term bleeding risks associated with anticoagulation.

The patient was subsequently seen by the electrophysiologist. Contributing factors to her AF were assessed to include hypertension, obesity, and possible undiagnosed sleep apnea. She was referred for a sleep study. Regarding the management of her AF, the options of antiarrhythmic drugs or catheter ablation were discussed. The patient expressed interest in pursuing catheter ablation. A second discussion addressed the use of an LAAC device to avoid the need for long-term anticoagulation, despite her elevated CHA₂DS₂-VASc score. The plan was to consider this option following further neurologist input, as a step separate from the ablation therapy. The patient continued on apixaban for anticoagulation. Catheter ablation had not been performed at the time of this report.

## Discussion

Moyamoya disease is a rare cerebrovascular condition characterized by chronic progressive stenosis or occlusion of the distal bilateral internal carotid arteries and the proximal segments of the anterior and middle cerebral arteries. This limits blood flow to the brain and puts patients at increased risk of stroke. To compensate, a new collateral network of small blood vessels develops. These vessels are thin-walled and structurally abnormal, making them susceptible to rupture and resulting in a high risk of intracranial hemorrhage. Moyamoya disease has a bimodal age distribution, typically presenting in childhood around age 5 and in adulthood between the third and fourth decades of life. The prevalence of Moyamoya disease is approximately 10.5 per 100,000, and the annual detection rate is 0.94 per 100,000 [5]. For unknown reasons, it is more common in females. The disease follows an autosomal dominant inheritance pattern with reduced penetrance [6].

AF is a common arrhythmia associated with an increased risk of embolic events due to thrombus formation and detachment in the left atrium and left atrial appendage. For patients with AF, anticoagulation is a cornerstone of stroke risk reduction. Non-vitamin K oral anticoagulants (NOACs), also known as DOACs, are the preferred agents for stroke prevention in this population over vitamin K antagonists (VKAs) or heparins [7]. Unfortunately, in patients with Moyamoya disease, the long-term use of anticoagulants may increase the risk of hemorrhagic transformation. According to the 2023 ACC/AHA/HRS guidelines, the decision to initiate anticoagulation in patients with AF should be based on individualized stroke risk assessment. The CHA₂DS₂-VASc score remains a useful tool, and anticoagulation is generally recommended for patients with a score of ≥2 in men and ≥3 in women, reflecting a high absolute annual risk of stroke [7].

When AF is complicated by Moyamoya disease, long-term use of NOACs increases the risk of hemorrhage, so other treatment options are often considered [4]. In some cases, the use of antiplatelet agents over anticoagulants may be preferred due to the bleeding risk associated with fragile collateral vessels. Drugs such as aspirin or clopidogrel may be used to reduce thromboembolic risk without the added bleeding risk of anticoagulants. In certain scenarios where anticoagulation is deemed necessary (e.g., high stroke risk due to AF), close monitoring is essential, and low-molecular-weight heparin or warfarin with a lower INR target may be considered.

LAAC is a procedure that has developed rapidly in recent years. The 2023 ACC/AHA AF guidelines recommend that in patients with AF who are at moderate to high risk of stroke but also have an increased risk of major bleeding from oral anticoagulation, percutaneous LAAC may serve as a reasonable alternative [7]. After successful LAAC, only short-term anticoagulation is typically required [8]; however, in high-risk patients, such as those with both Moyamoya disease and AF, single antiplatelet therapy (SAPT) is considered a safe and practical alternative commonly used in clinical practice [9,10]. There are currently limited cases and relevant studies of LAAC in patients with Moyamoya disease. This case discusses the challenges in managing anticoagulation in patients with concurrent Moyamoya disease and AF.

## Conclusions

Given the complexity of managing patients with both Moyamoya disease and paroxysmal AF, treatment should be individualized. This case uniquely illustrates the challenge of balancing the risk of thromboembolism against the heightened risk of intracranial hemorrhage in a patient with fragile and vulnerable cerebral vasculature. Careful consideration of the benefits and risks of anticoagulation versus antiplatelet therapy is essential, with an emphasis on preventing stroke while minimizing bleeding risk. Further research and additional case reports, such as the one described, are needed to help inform best practices in these rare and complex clinical scenarios.
